# Neuropilin-1 predicts poor prognosis and promotes tumor metastasis through epithelial-mesenchymal transition in gastric cancer

**DOI:** 10.7150/jca.52851

**Published:** 2021-04-30

**Authors:** Qianna Jin, Qianqian Ren, Xiaona Chang, Haixing Yu, Xin Jin, Xiaoming Lu, Nan He, Guobin Wang

**Affiliations:** 1 Department of Radiology, Union Hospital, Tongji Medical College, Huazhong University of Science and Technology, 1277 JieFang Avenue, Wuhan 430022, China.; 2 Cancer Center, Union Hospital, Tongji Medical College, Huazhong University of Science and Technology, 1277 JieFang Avenue, Wuhan 430022, China.; 3 Department of Pathology, Union Hospital, Tongji Medical College, Huazhong University of Science and Technology, 1277 JieFang Avenue, Wuhan 430022, China.; 4 Department of Gastrointestinal Surgery, Union Hospital, Tongji Medical College, Huazhong University of Science and Technology, 1277 JieFang Avenue, Wuhan 430022, China.; 5 Hubei Province Key Laboratory of Molecular Imaging

**Keywords:** NRP1, Stomach neoplasms, Prognosis, EMT, Oncogenes

## Abstract

We aimed to determine whether Neuropilin-1 (NRP1) promotes gastric cancer (GC) metastasis by inducing epithelial-mesenchymal transition (EMT), and to clarify its regulatory mechanism. Using the data of GC patients in The Cancer Genome Atlas (TCGA) and Gene Tissue Expression (GTEx) databases, combined with the data of GC patients in our medical center, the effect of NRP1 on the prognosis of GC patients were analyzed. Then, we investigated the role of NRP1 in GC metastasis and its potential mechanism. The level of NRP1 was up-regulated in GC tissues and associated with poor prognosis of GC patients. The expression of NRP1 was closely related to maximum tumor diameter, invasion depth, lymphnode metastasis, distant metastasis, and advanced TNM stage, and was an independent prognostic factor for overall survival (OS) in GC patients. Besides, the results of *in vitro* indicated that NRP1 could induce EMT to promote the migration and invasion of GC cells by activating PI3K/Akt signaling pathway, and the HGF/c-Met axis was involved in this process. This study determined that NRP1 was a gene that promotes gastric cancer. NRP1 induced EMT to enhance the migration and invasion ability of GC cells by activating PI3K/Akt signaling pathway. NRP1 was an independent prognostic marker for OS in GC patients and expected to be a therapeutic target for GC patients.

## Introduction

Gastric cancer (GC) is one of the main causes of cancer-related deaths and poor 5-year survival of patients worldwide 1, 2. More than 90% of the mortality in GC patients is associated with cancer metastasis 3. Therefore, investigating the metastasis mechanism is crucial for improving the prognosis of GC patients.

Several studies have found that epithelial-mesenchymal transition (EMT) is a sign of malignant tumor progression 4, 5. During EMT process, epithelial cells lose epithelial phenotypes such as cell polarity, the ability of connecting to the basement membrane, etc, and obtain interstitial phenotypes such as migration and invasion, anti-apoptosis, and the ability to degrade extracellular matrix. EMT is a dominant biological process for malignant tumor epithelial cells to acquire the ability of migration and invasion. Accordingly, elucidating the molecular mechanism that regulates the EMT process of cancer cells, clarifying their pathological significance in the occurrence, development, and metastasis of malignant tumors, exploring diagnostic and therapeutic methods based on EMT biomarkers are key scientific issues to study tumor metastasis mechanism.

Neuropilin-1(NRP1) is a transmembrane glycoprotein that acts as a co-receptor for semaphorin (SEMA) and vascular endothelial growth factor and is a key regulator of nervous system development, angiogenesis, immunity, and tumorigenesis 6-9. NRP1 is found to be abnormally expressed in many cancers, including GC, breast cancer, and esophageal cancer, and its abnormally expression indicates poor prognosis and cancer metastasis 10-13. NRP1 is considered to regulate tumor migration, invasion, and angiogenesis as a receptor for some cancer factors or interacting with certain signaling pathways (ERK1/2, P38 MAPK, Stat5, and Akt, etc) 14-16. Nevertheless, whether NRP1 promotes GC metastasis by inducing EMT and its regulatory mechanism remain unclear. In this study, we demonstrated that the level of NRP1 was closely related to poor prognosis of GC patients. More importantly, we found that NRP1 could induce EMT to promote the invasion and migration of GC cells by activating the PI3K/Akt signaling pathway. Our study revealed that NRP1 played a vital role in the invasion and metastasis of GC cells by inducing EMT and provided a theoretical basis for determining NRP1 as an effective biomarker and therapeutic target for GC.

## Materials and Methods

### Patients and specimens

The study included 210 GC patients who underwent surgical resection in the General Surgery of Union Hospital (Huazhong University of Science and Technology, Wuhan, China) from June 2013 to June 2014. These patients did not receive preoperative chemotherapy or radiotherapy, and there were no severe heart, lung, or kidney comorbidities. All patients were diagnosed with a pathological confirmation. Tissue samples were obtained from excised specimens, including tumor tissues and peritumoral normal mucosal tissues (more than 5cm from the edge of the tumor), quickly frozen in liquid nitrogen, and stored at -80℃ until use. The clinicopathologic data including age, gender, tumor size (maximum diameter), tumor location, the degree of tumor differentiation, preoperative carcinoembryonic antigen (CEA), and TNM stage were collected from the patient's medical history. Tumor stage was determined according to the 8th edition of the American Joint Committee on Cancer (AJCC) TNM classification system. The median follow-up period for the surviving patients was 55.4 months (range 3-65 months). Overall survival (OS) was defined as the interval between surgery and death, or between surgery and the last observation date for surviving patients.

This study was carried out according to the Code of Ethics of the World Medical Association (Declaration of Helsinki) and was approved by the research ethics committee of Union hospital, Huazhong University of Science and Technology, China (No. 2018LSZS085). Informed consent was obtained from all patients in this study.

### Public data analysis

All the work on GC's data from TCGA and GTEx database was carried out by using the online tool Gene Expression Profiling Interactive Analysis (GEPIA, http://gepia.cancerpku.cn/index.html) 17. GEPIA performed survival analysis on gene expression levels and required a log-rank test for hypothesis assessment.

### IHC staining and evaluation

Immunohistochemistry (IHC) staining was performed. All specimens were fixed in 4% formaldehyde solution, embedded into paraffin, and then cut into 4μm sheets. In the next step, the tissue slides were dewaxed with xylene, dehydrated by using gradient alcohol and retrieved antigen by microwave. NRP1 antibody (Abcam, Cambridge, USA) diluted to 1:200. The secondary antibody was labeled with peroxidase and developed with diaminobenzidine. The staining procedure was carried out according to the manufacturer's instructions. The result of immunohistochemistry (IHC) was judged by two experienced pathologists and quantified by using a semi-quantitative comprehensive scoring method based on the intensity of staining and the ratio of stained cells 18: the immunoreactive score (IRS) depended on the intensity of staining (SI) and the percentage of the positive cell (PP), IRS=SI+PP. SI: 0 points for the colorless, 1 point for the light yellow, 2 points for the brownish yellow, and 3 points for the tan. PP: 0 points for negative, 1 point for positive cells ≤10%, 2 points for 11% to 50%, 3 points for 51% to 100%. IRS: 0 negatives, ≤3 low expressions, > 3 high expressions.

### Cell culture and establishment of stably transfected cell lines

Five GC cell lines (SGC-7901, AGS, MGC-803, BGC823, and MKN45) and immortalized gastric mucosal cell line (GES-1) were obtained from the Cell Center of the Chinese Academy of Sciences (Shanghai, China). These cells were cultured in the recommended medium supplemented with 10% fetal bovine serum and incubated in 5% CO_2_ at 37℃. SGC-7901 cells with lower endogenous NRP1 expression were selected for transfecting with NRP1 overexpression plasmids (pCDNA3.1 (1) -NRP1) or empty vectors (pCDNA3.1 (1)) (Invitrogen, California, USA). Lipofectamine 2000 (Invitrogen, California, USA) was used as the transfection reagent according to the manufacturer's operating manual. Transfected SGC-7901 cells were selected with 600 mg/mL G418 (Invitrogen, California, USA). The transfection efficiency on cell lines with NRP1 over-expression plasmid or empty vector was evaluated by Western blotting and qRT-PCR.

### RNA interference

Small interfering RNA (siRNA) oligonucleotides specific to NRP1 and siRNA control oligonucleotides were obtained from RiboBio Co. Ltd (Guangzhou, China). MGC-803 cells (1×10^5^) with NRP1 higher endogenous expression were cultured in 6-well plates until 50% confluence was reached, and then they were transfected with 100 nM of the indicated siRNA using riboFECT™ CP Reagent (RiboBio, Guangzhou, China) according to the manufacturer's instructions. The efficiency of NRP1 knockdown was analyzed by Western blotting and qRT-PCR after 48 hours.

### RNA isolation and quantitative real-time polymerase chain reaction

Total RNA was extracted using RNeasy Mini Kit (Qiagen, California, USA). RNA was subsequently reverse transcribed to generate complementary DNA (cDNA) with a first-strand cDNA Synthesis Kit (Thermo Fisher, California, USA). Quantitative reverse transcription-polymerase chain reaction (qRT-PCR) was performed to assess NRP1 and β-actin expression with LightCycler 480 SYBR Green I Master (Roche, Basel, Switzerland). The level of β-actin was used for normalization. The PCR primers used were as follows: NRP1 (sense: TGTGCCAAAGATGTCAGAGA, antisense: ACCTGGTGTTTTCTGTCCAC) and β-actin (sense: CAATGAGCTGCGTGTGGCT, antisense: TAGCACAGCCTGGATAGCAA).

### Immunofluorescence

Cells were seeded at 5×10^5^ cells per well in a 24-well plate with a 12-mm round poly-lysine-coated glass coverslip. Cells after 4% paraformaldehyde fixation, coverslips were washed three times in PBS and then blocked with 5% BSA in PBS with 0.3% Triton X-100 for 1h at room temperature with gentle shaking, followed by incubation with anti-E-cadherin antibody (1:50 in 1% BSA, Cell Signaling Technology, USA) and anti-vimentin (1:50 in 1% BSA, Cell Signaling Technology, USA) overnight with gentle shaking at 4°C. Coverslips were then washed PBS and incubated for 1 h at room temperature with secondary anti-rabbit Alexa Fluor 594 (diluted 1:200 in 1% BSA). Coverslips were washed three times with PBS, incubated with a solution of DAPI (1:5000), and TO-PRO (1:1000) in water for 10 minutes to stain the nuclei, and imaged.

### Western blotting

Total protein was isolated from the cells and defined the concentration by using Coomassie Protein Assay (Thermo Fisher Scientific, USA). The protein sample from the previous step was separated by SDS-PAGE and transferred to polyvinylidene difluoride (PVDF) membrane. After blocking for 60 minutes in skim milk at room temperature, the membrane was incubated with specific primary antibody overnight at 4°C. The next day, the membrane was incubated with a secondary antibody (1:5000 dilution, Cell Signaling Technology, USA) for 2 hours at room temperature. β-actin was served as a control. The signal on the membrane was detected and imaged by the Odyssey Infrared Imaging System (LI-COR, Nebraska, USA). The expression of all proteins was quantified by Image J software (https://imagej.net) and normalized to the quantified value of β-actin. The following antibodies were used in western blotting experiments, e.g. NRP1 (1:1000, Abcam, Cambridge, USA), E-cadherin, N-cadherin, Vimentin, Snail, Twist, β-actin (1:1000, Cell Signaling Technology, USA).

### Cell proliferation assay

Cell proliferation was determined using the Cell Counting Kit-8 (CCK-8) assay (Dojindo, Kumamoto, Japan). Tested cells were seeded into 96-well plates (Corning, New York, USA) at a density of 1,000 cells/well. Cells could grow for 1, 2, 3, 4, 5, 6, and 7 days, and then 10μl of CCK-8 solution was added to each well and incubated at 37℃ for 4 h. Cell viability was detected by measurement of absorbance at 490nm.

### Transwell migration and invasion analysis

Transwell migration and invasion analysis were performed using a 24-well modified Boyden chamber (Greiner Bio-One GmbH, Frickenhausen, Germany) with or without Matrigel (BD Biosciences, NJ, USA) coating, respectively. Cells (1×10^5^) were seeded into the upper chambers in serum-free medium and fetal bovine serum as a chemoattractant. After 48h of incubation at 37℃ in 5%CO_2_, migrated and invaded cells were quantified by dissolving cell-bound crystal violet in 10% acetic acid, and absorbance was measured at 540nm.

### Enzyme-linked immunosorbent assays (ELISA)

Protein expression of HGF, EGF, FGF, and VEGF in the culture medium of colon cancer cell lines collected from above experiments was measured by ELISA kits (Abcam, Cambridge, MA, USA).

### Statistical analysis

Statistical analyses were performed with SPSS 25.0 (SPSS Inc, Chicago, USA) and Prism software (GraphPad, California, USA). Pearson's chi-squared test and Fisher's exact test were applied for categorical variables; continuous variables were analyzed by the Student's *t* test. Survival and univariate analysis were determined by Kaplan-Meier analysis. The Cox proportional hazards regression model was applied to perform a multivariate analysis. All statistical analyses were two-sided, and *P* value < 0.05 was considered statistically significant.

## Result

### Upregulation of NRP1 expression in GC tissue

**Figure 1:**
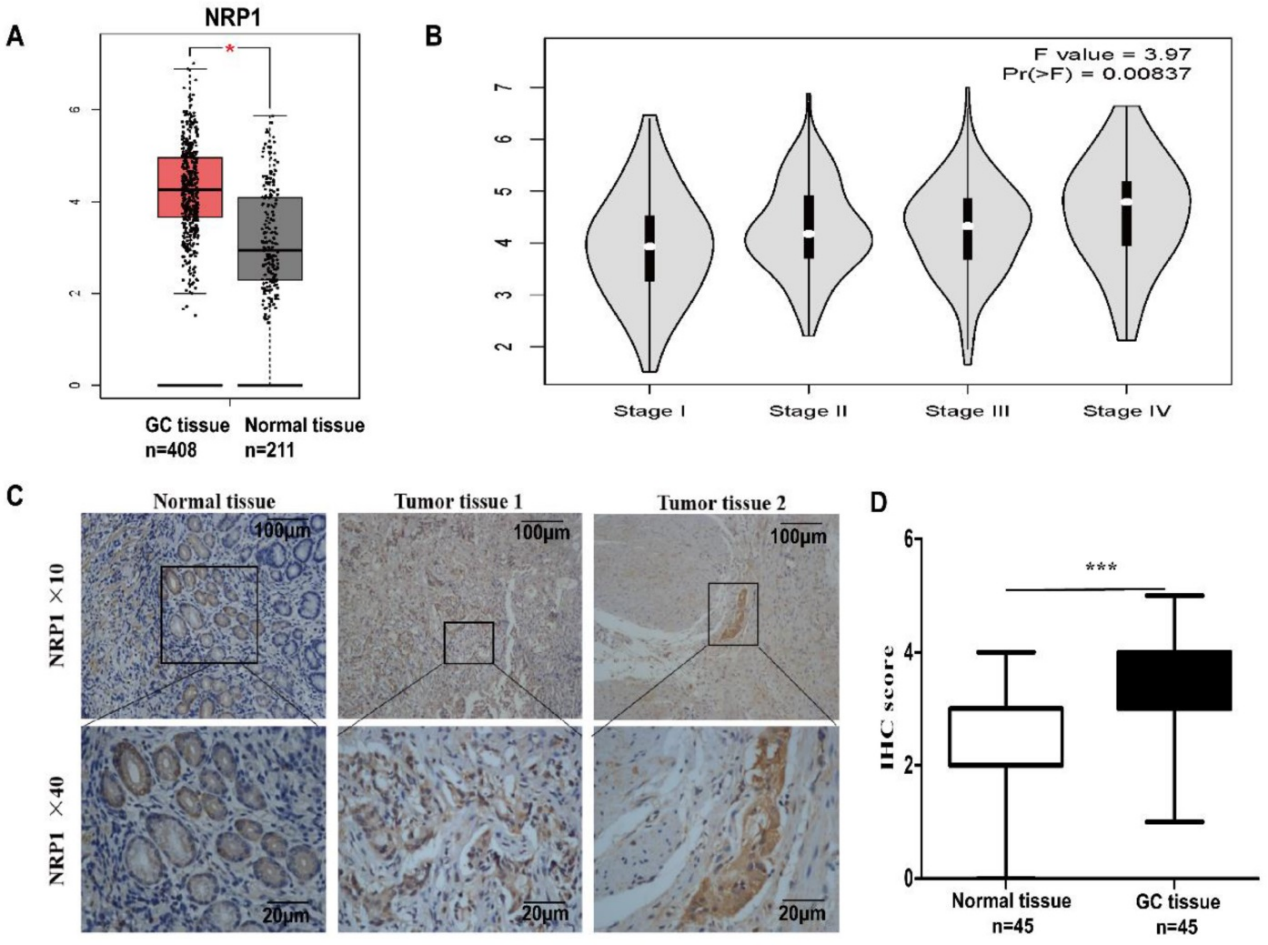
** NRP1 upregulated in GC tissue and linked with AJCC stage.** (A) The GEPIA results revealed that NRP1 expression was significantly upregulated in the GC tissues. (B) The level of NRP1 was linked with the pathological stage of the tumor. The level of NRP1 in patients with stage III and IV was significantly higher than that of patients with stage I and II. (C) The representative image of NRP1 expression in GC tissues was detected by IHC. Scale bars were indicated. (D) The IHC staining score of NRP1 protein in GC tissues and paired normal mucosal tissues. GC, gastric cancer; NRP1, Neuropilin-1; IHC, Immunohistochemistry; *, *P*< 0.05; **, *P*< 0.01; ***, *P*< 0.001; ns, no significant.

To reveal the expression of NRP1 in GC, the online tool GEPIA was performed to analyze the data of GC patients in the TCGA and GTEx databases. It was shown that the level of NRP1 mRNA in GC tissue was significantly higher than that in normal tissue (*P*<0.05, Fig. 1A), and significantly correlated with the pathological stage of the tumor (F=3.97, Pr (>P) =0.00837, Fig. 1B). The level of NRP1 in stage III and IV patients was significantly higher than that in stage I and II. Furthermore, the NRP1 protein levels were also examined in a group of 45 GC tissues paired with adjacent normal mucosa tissues by IHC. The result of IHC revealed that the specific NRP1 staining was detected in the cytoplasm and cell membrane of malignant epithelial cells (Fig. 1C). The IHC staining score of NRP1 in GC tissues was higher than that of adjacent normal mucosa tissues (3.17±0.1598 *vs.* 1.87±0.13, respectively;* P*<0.001, Fig. 1D).

### High NRP1 expression was an independent predictor of poor prognosis in patients with gastric cancer

To explore the clinical significance of NRP1 expression in GC, we performed immunohistochemical analysis on 210 GC samples to examine the expression of NRP1 protein. These cases included 27 patients with distant metastasis, who were diagnosed with distant metastasis by preoperative examination or intraoperative tissue biopsy. According to the aforementioned IHC scoring criteria, 120 of 210 GC patients (57.1%) were defined as high NRP1 protein expression, while the remaining 90 patients (42.9%) were classified as low NRP1 protein expression. We pooled the NPR1 level of GC tissue with patients' clinicopathologic factors (age, sex, tumor size, tumor site, tumor differentiation, TNM stage, depth of tumor invasion, local lymph node metastasis, distant metastasis, and preoperative CEA) for statistical analysis. Statistical results showed that the level of NRP1 in GC was significantly related to tumor size, TNM stage, depth of tumor invasion, N stage, and distant metastasis (*P*<0.001), but was not significantly related to other clinicopathologic factors (Table 1).

**Figure 2:**
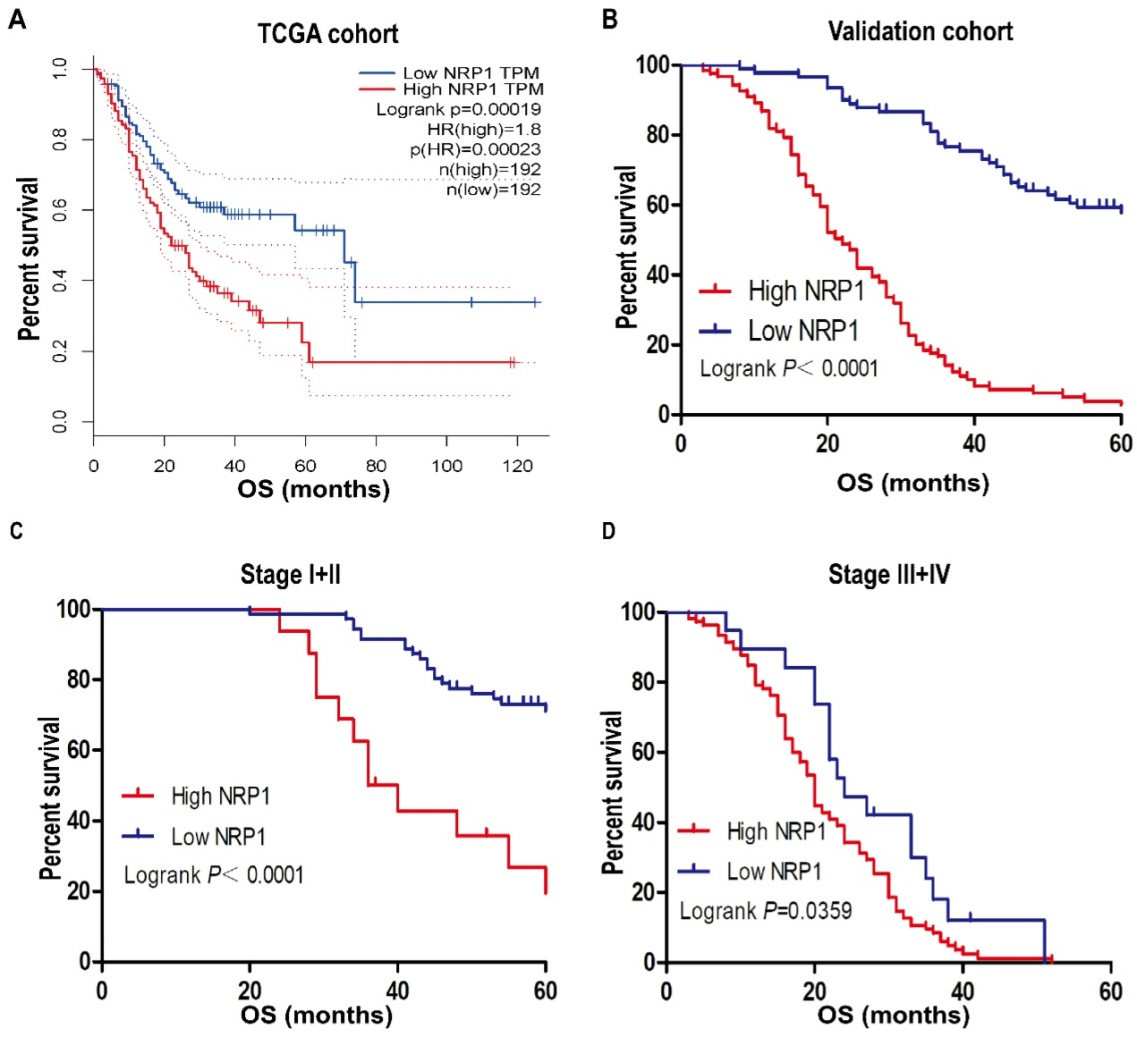
** NRP1 expression was associated with OS.** (A) Kaplan-Meier survival analysis of OS in TCGA cohort. (B) Kaplan-Meier survival analysis of OS in 210 gastric cancer patients (validation cohort). Stratified analysis (C) the OS of Stage I+II GC patients and (D) the OS of Stage III+IV GC patients. OS, overall survival; TCGA, The Cancer Genome Atlas. *, *P*< 0.05; **, *P*< 0.01; ***,* P*< 0.001; ns, no significant.

In our study of 210 GC patients, 120 patients with high NRP1 expression had shorter OS than those with low NRP1 expression by using Kaplan-Meier analysis (*P*<0.001, Fig. 2B), the result was consistent with the analysis of GC patients from the TCGA database by using online tool GEPIA (*P*<0.001, Fig. 2A). Stratified analysis results also showed that the prognosis of GC patients with high NRP1 expression was worse than those with low NRP1 expression in stage I+II and stage III+IV group (*P*<0.05, Fig. 2C, D). Univariate analysis was employed to estimate the clinical factors which influenced the OS of GC patients. As described in Table 2, high NRP1 expression was identified as a risk factor of poor prognosis (HR, 7.056; 95%CI, 4.691-10.614; *P*<0.001). Furthermore, tumor size (HR, 4.254; 95%CI, 3.013-6.006; *P*<0.001), TNM stage (HR, 8.855; 95%CI, 6.402-12.248; *P*<0.001), tumor invasion depth (HR, 14.095; 95%CI, 7.485-26.540; *P*<0.001), lymph node metastasis (HR, 6.72; 95%CI, 4.073-11.086; *P*<0.001), and distant metastasis (HR, 29.549; 95%CI, 16.592-52.625; *P*<0.001) were also risk factors for OS. Towards further identifying the independent prognostic factors for OS in GC patients, multivariate Cox regression analysis was performed on all OS risk factors determined by univariate analysis. As shown in Table 2, NRP1 expression (HR, 2.122; 95%CI, 1.362-3.305; *P*=0.001), TNM stage (HR, 4.032; 95%CI, 2.416-6.73; *P*<0.001), and distant metastasis (HR, 3.569; 95%CI, 1.669-7.63; *P*=0.001) were identified as an independent prognostic factor for OS in GC patients.

### NRP1 promoted the proliferation, invasion, and migration of GC cells

**Figure 3:**
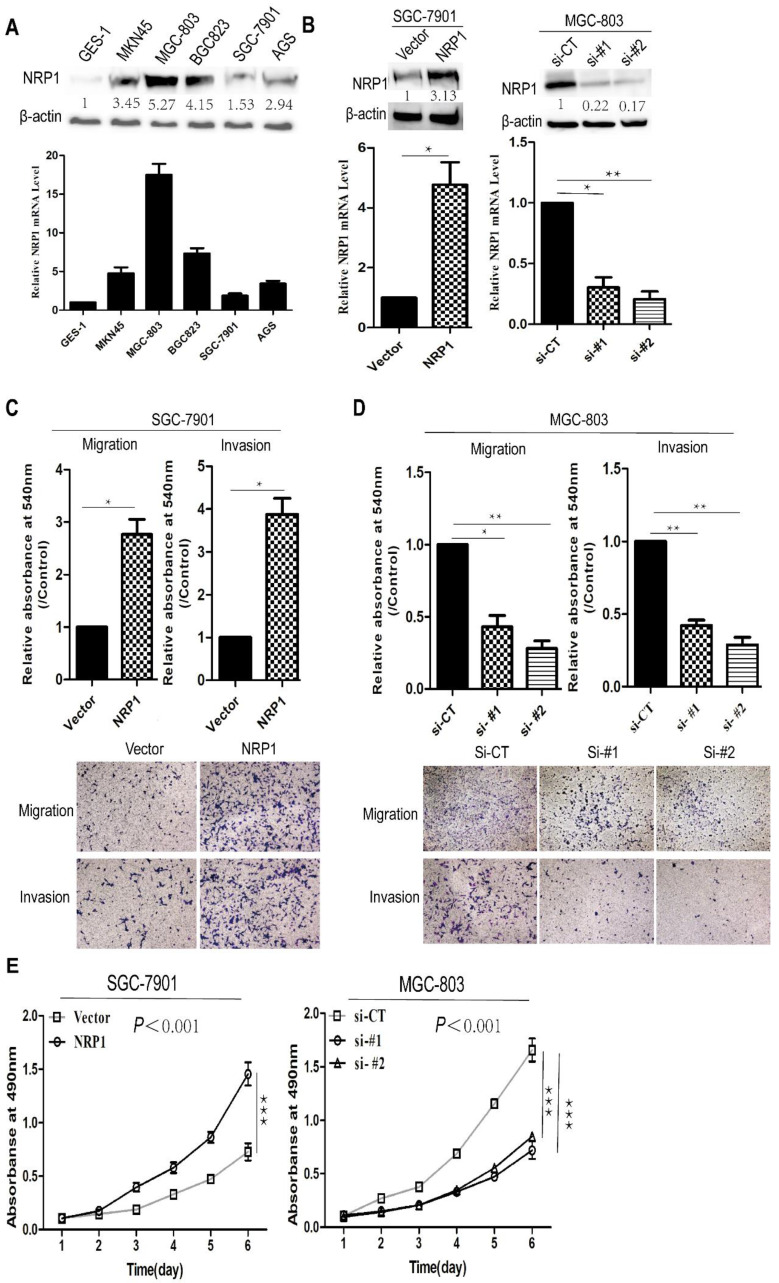
**NRP1 overexpression promoted proliferation, migration and invasion in GC cells.** (A) The expression of NRP1 in 5 GC cell lines (SGC-7901, MGC-803, BGC823, AGS, and MKN45) and 1 immortalized gastric mucosa cell, GES-1, were analyzed by western blotting (top panel) and qRT-PCR (bottom panel), respectively. (B) The expression of NRP1 in SGC-7901 cells transfected with vector or NRP1 overexpression plasmid (Left panel) and MGC-803 cells with NRP1-specific siRNA (Right panel) were detected by western blotting and qRT-PCR. (C) Transwell migration and matrigel invasion assays were conducted in SGC-7901 cells transfected with vector and NRP1 overexpression plasmid. Data were mean ± SD. (D) Transwell migration and matrigel invasion assays were conducted in MGC-803 cells with control siRNA (siCT) or two NRP1-specific siRNA (si#1/si#2). Data were mean ± SD. (E) Cell proliferation was evaluated by the CCK-8 assay. These data was obtained from three independent experiments. GC, gastric cancer; NRP1, Neuropilin-1; *, *P*< 0.05; **, *P*< 0.01; ***,* P*< 0.001; ns, no significant.

To determine the function of NRP1 in the progression of GC, we examined the levels of NRP1 in five human GC cell lines (MGC-803, BGC823, SGC-7901, MKN45, AGS) and immortalized gastric mucosal cell line (GES-1) through qRT-PCR and Western-blotting. It was shown that NRP1 was significantly higher expressed in GC cell lines than in GES-1, and NRP1 expressed differently in our group of 5 GC cell lines (Fig. 3A). SGC-7901 cells with low NRP1 endogenous expression were transfected with NRP1 overexpression plasmid, MGC-803 cells with NRP1 high endogenous expression were transfected with small interfering RNA (siRNA) specific to NRP1. After transfection, the level of NRP1 was confirmed by western-blotting and qRT-PCR (Fig. 3B).

Subsequently, a transwell assay was conducted to evaluate the effect of NRP1 overexpression or knockdown on GC cell migration and invasion. SGC-7901 cells with NRP1 overexpression exhibited more aggressive potential in migration and invasion (*P*=0.024 and *P*=0.016, Fig. 3C). In contrast, MGC-803 cells with siRNA-NRP1 showed significantly decreased migrating and invading cells than control group (siCT) (*P*<0.05, Fig. 3D). CCK-8 assay was used to examine cell proliferation. Results showed that NRP1 overexpression significantly increased GC cell proliferation (*P*<0.001), while NRP1-knockdown inhibited GC cell proliferation (*P*<0.001, Fig. 3E). In brief, NRP1 could promote the proliferation, invasion, and migration of GC cells.

### NRP1 induced EMT in GC cells

**Figure 4:**
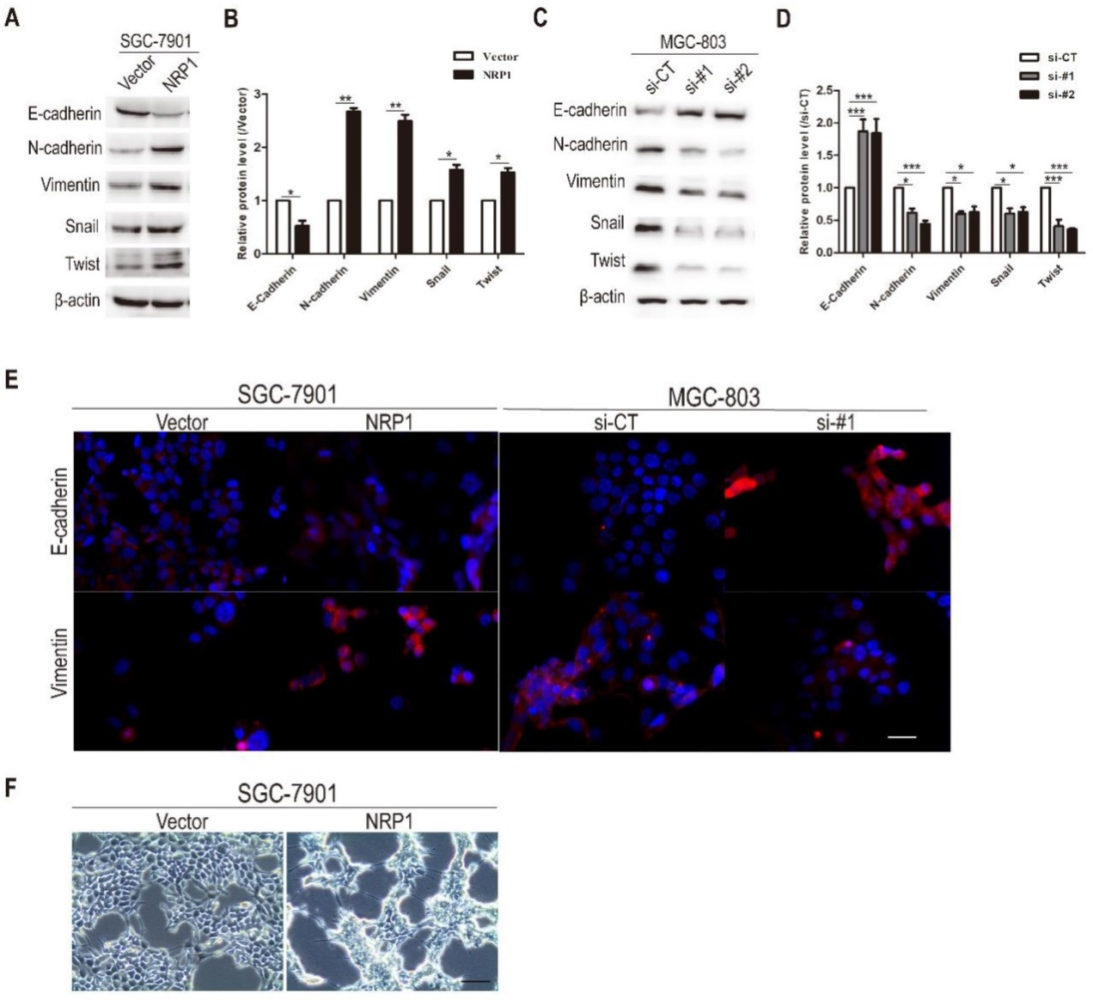
** NRP1 promoted EMT in GC cells.** (A-B) The expression of epithelial markers (E-cadherin), mesenchymal marker (Vimentin and N-cadherin), and transcriptional factors (Snail and Twist) were examined by western-blotting in SGC-7901 cells with NRP1-overexpressing plasmid. (C-D) The expression of epithelial markers (E-cadherin), mesenchymal marker (Vimentin and N-cadherin), and transcriptional factors (Snail and Twist) were examined by western-blotting in MGC-803 cells with NRP1-siRNA. (E) Immunofluorescence staining of SGC-7901 cells with vector or NRP1-overexpress plasmid, and MGC-803 cells with control or NRP1-siRNA. E-cadherin and Vimentin were red, the nuclei of the cells were blue. (F) NRP1-overexpressing in SGC-7901 cells induced EMT with morphological transformation and alterations in cellular configuration. Scale bars, 50μm (E), 200μm (F). These data were obtained from three independent experiments. GC, gastric cancer; NRP1, Neuropilin-1; *, *P*< 0.05; **,* P*< 0.01; ***, *P*< 0.001; ns, no significant.

Previous studies had proposed that EMT was associated with the migration, metastasis, and progression of cancer cells. Therefore, we investigated the effects of NRP1 overexpression or knockdown on EMT in GC cells. In the first step, western-blotting analysis was conducted to examine the effect of NRP1 on EMT-related hallmarks and transcriptional factors 33, 34. Compared with the control group, epithelial markers E-cadherin was down-regulated in SGC-7901 cells with NRP1 overexpression plasmid, while mesenchymal markers (Vimentin, N-cadherin) and transcriptional factors (Snail, Twist) were up-regulated (*P*<0.05, Fig. 4A-B). In contrast, MGC-803 cells with siRNA-NRP1 had reduced the expression of N-cadherin, Vimentin, Snail, and Twist compared to control cells, while the expression of epithelial markers E-cadherin was increased (*P*<0.05, Fig. 4C-D). Immunofluorescence staining results also showed that NRP1 overexpression linked with reducing the expression of E-cadherin and increasing the expression of Vimentin. In contrast, the up-regulation of E-cadherin and down-regulation of Vimentin were associated with NRP1 knockdown (Fig. 4E). Under the microscope, SGC-7901 cells with overexpressed NRP1 exhibited a representative EMT morphology (loose cell contact and spindle-shaped cell morphology) (Fig. 4F). In brief, NRP1 could induce EMT in GC cells.

### NRP1 induced EMT and promoted the migration and invasion of GC cells by activating the PI3K/Akt pathway

**Figure 5:**
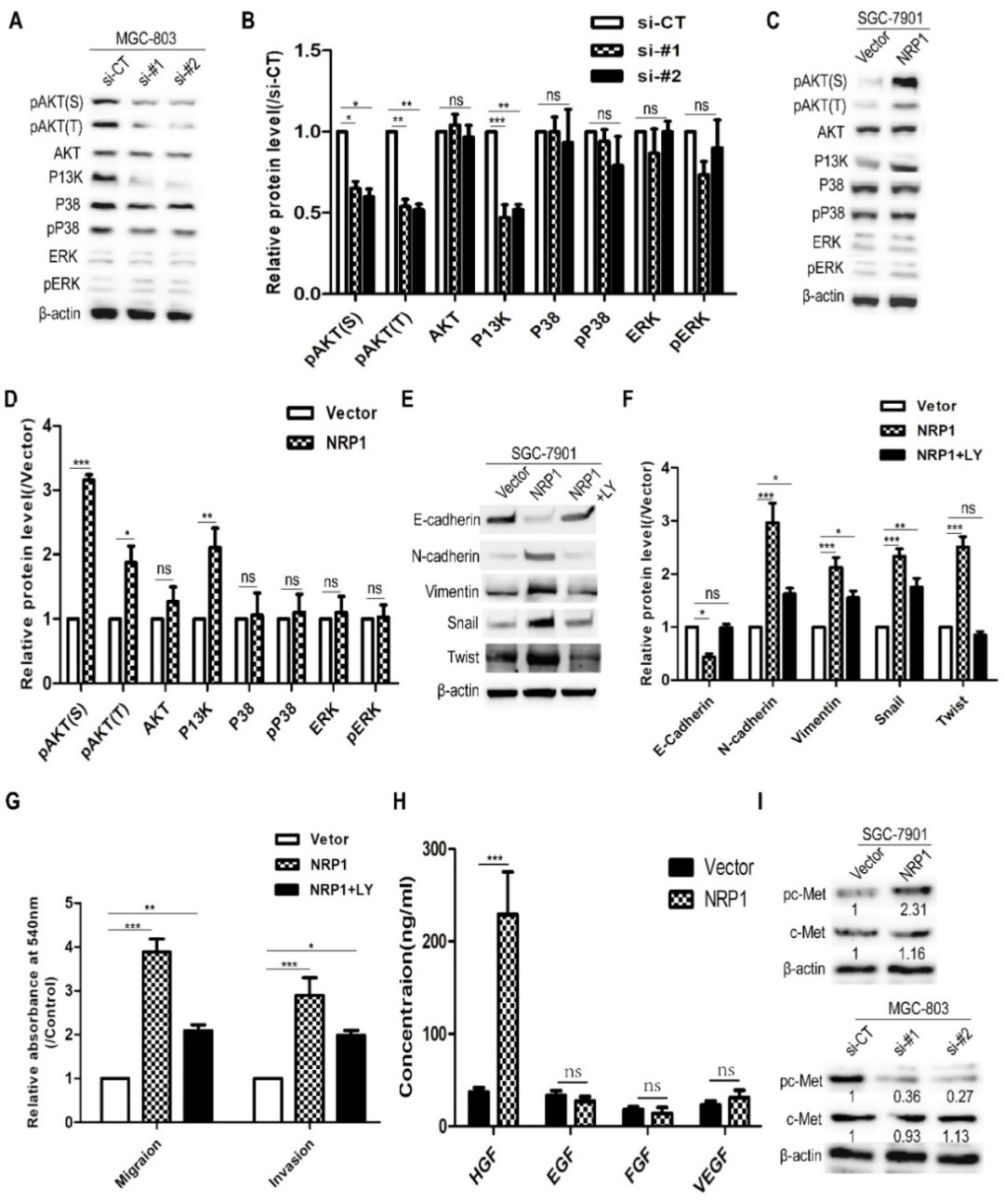
** NRP1 induced EMT to promote the migration and invasion of GC cells by activating the PI3K/Akt pathway.** (A-B) The effect of highly NRP1 expression on Akt, P38, ERK and PI3K signaling pathways was assessed by Western blotting. (C-D) The effect of NRP1 knockdown on Akt, P38, ERK and PI3K signaling pathways was assessed by western blotting. (E-F) SGC-7901 cells with NRP1 overexpression plasmid were treated with LY294002, and the expression of E-cadherin, N-cadherin, Vimentin, Snail and Twist were detected by western-blotting. (G) SGC-7901 cells with NRP1 overexpression plasmid were treated with LY294002 and applied to transwell analysis. (H) The levels of several extracellular growth factors (HGF, EGF, FGF, and VEGF) in the cell culture supernatants were measured by ELISA. (I) c-Met protein levels in GC cells transfected with siNRP1 and NRP1 overexpression plasmid was detected by Western-blotting. Data were mean ± SD. These data were obtained from three independent experiments. GC, gastric cancer; NRP1, Neuropilin-1; LY294002, a PI3K/Akt signaling pathway inhibitor; *, *P*< 0.05; **, *P*< 0.01; ***, *P*< 0.001; ns, no significant.

To explore the mechanism of NRP1 induced EMT to promote the migratory and invasive ability of GC cells, western-blotting analysis was used to detect several common cancers metastasis-related signaling pathways (e.g. Akt, P38, ERK, and PI3K) 3. It was suggested that MGC-803 cells with siRNA-NRP1 could inhibit Akt and PI3K activation (*P*<0.05, Fig. 5A-B). In contrast, SGC-7901 cells with NRP1 overexpression plasmid can significantly increase the levels of phosphorylated Akt (Ser473 and Thr 308) and PI3K (*P*<0.05, Fig. 5C-D). These results indicated that NRP1 could enhance Akt phosphorylation and P13K activation. To further determine whether the PI3K/Akt pathway was involved in the above effect of NRP1, SGC-7901 cells were treated with LY294002 (50nM, Cell Signaling Technology, USA) for 12h, a PI3K/Akt pathway inhibitor. It was shown that LY294002 could attenuate the changes in the expression of EMT-related hallmarks (E-cadherin, Vimentin, and N-cadherin) and transcription factors (Snail, Twist) caused by NRP1 overexpression (Fig. 5E-F). Also, the result of the transwell assay showed that LY294002 could partially reverse the promoting effect of NRP1 overexpression on the migratory and invasive ability of GC cells (Fig. 5G).

To investigate how NRP1 activates the PI3K/AKT pathway (eg. ligands that were involved). The levels of several extracellular growth factors (HGF, EGF, FGF, and VEGF) in the cell culture supernatants were measured by ELISA. In 48-h cell culture, SGC-7901 transfected with NRP1 overexpression plasmid secreted more HGF into serum-free medium than other growth factors (Fig. 5H). c-Met is known to be the ligand of HGF, and its phosphorylation activates downstream signaling 26, 40. Therefore, we examined c-Met protein levels in GC cells transfected siRNA-NRP1 and overexpressed NRP1 plasmid. As shown in Fig. 5I, the expression of c-Met was not enhanced by NRP1 overexpression, but phosphorylation of c-Met was stimulated. Conversely, phosphorylation of c-Met was suppressed in siRNA-treated cells. Therefore, we believe NRP1 could induce EMT to promote the migration and invasion of GC cells by activating PI3K/Akt signaling pathway, and the HGF/c-Met axis was involved in this process.

## Discussion

NRP1, a transmembrane glycoprotein, was originally involved in axon guidance and angiogenesis based on interaction with semaphorin and VEGF family 19. Moreover, NRP1 was found to promote tumor progression via interacting with various extracellular growth factors and their receptors, including hepatocyte growth factor (HGF) and its receptor (cMet), fibroblast growth factors (FGFs), vascular endothelial growth factor (VEGF), transforming growth factor (TGF-β), etc 12, 20, 21. NRP1 could promote tumor metastasis through several mechanisms, such as facilitate endothelial cell migration induced by VEGF 22-24, boost the migration and proliferation of tumor cells through autocrine Semaphorin 3A (Sema 3A) 25 and HGF/SF signaling 26, stimulate tumor growth by increasing fibronectin fibril assembly in the tumor microenvironment 27, uphold dedifferentiation and propagation phenotypes of cancer cells 28, maintain the properties of cancer stem cells 29, and induce tumor immunosuppression signaling 8, 30. Furthermore, NRP1 had been shown to enhance EMT in oral squamous cell carcinoma and non-small cell lung cancer, thereby promoting cancer cell invasion and metastasis 31, 32.

EMT was the initial step of tumor cell invasion and metastasis. It manifested in the loss of cell polarity and cell adhesion to promote tumor cell invasion and metastasis 33. EMT linked with a series of molecular events which included down-regulation and dysfunction of E-cadherin, relocalization of β-catenin from membrane to nucleus, and up-regulation of the mesenchymal marker proteins (e.g. N-cadherin, Vimentin) 34, 35. Besides, the increased expression of EMT-related transcription factors, including Twist, Snail, Zeb, and Slug, promoted the EMT process. Its mechanisms included reprogramming of tumor cells to obtain stem cell characteristics 36, leading to rapid tumor development and inefficient treatment 37, inhibiting tumor cell senescence and apoptosis 38, and promoting tumor cells metabolic changes 39. In this study, we explored that NRP1 promoted the invasion and metastasis of GC cells via inducing EMT and its related regulatory mechanisms.

We used the online tool GEPIA to analyze GC patients' data from the TCGA and GTEx databases. The results were: (1) The level of NRP1 in normal mucosa tissues was significantly lower than that of GC tissues (*P*<0.05); (2) The expression of NRP1 was closely related to the TNM stage of GC patients; (3) The OS of GC patients with high NRP1 expression was significantly lower than that of patients with low NRP1 expression (*P*=0.00019, HR=1.8). Afterwards, we verified the above research results by using our GC cohort. It was indicated that the highly expression of NRP1 was related to tumor maximum diameter greater than 5cm, deeper invasion, severe lymph node metastasis, distant metastasis, later TNM stage, and shorter OS. Combined with Cox proportional hazards regression model analysis, NRP1 was identified as one of the independent prognostic factors for OS in GC patients.

*In vitro*, the results of CCK-8 and Transwell assay showed that NRP1 promoted the proliferation, migration, and invasion of GC cell, which was consistent with previous research 13. Afterwards we examined the expression of EMT-related hallmarks and transcriptional factors. It was found that NRP1 with ectopic expression could induce EMT in GC cells and significantly enhance the migration and invasion ability of GC cells. In contrast, the migration and invasion ability of GC cells were decreased when EMT was inhibited by NRP1-siRNA. Further research demonstrated that the above-mentioned effects caused by NRP1 were achieved by activating the PI3K/Akt signaling pathway, and the HGF/c-Met axis was involved in this process. NRP1 could promote tumor progression through potentiating the activity of the HGF/SF autocrine c-Met signaling pathway 26. On HGF binding, c-Met undergoes dimerization and autophosphorylation of tyrosine residues, generating multidocking sites, which activates the intracellular PI3K/Akt signaling pathway 40. The activation of PI3K/Akt signaling pathway could promote the mesenchymal transformation of malignant tumor epithelial cells by upregulating the level of phosphorylation Twist which was a key transcription factor in the EMT process 41-43, inhibiting the degradation of Snail by promoting the ubiquitination of GSK-3β 44, and directly upregulating Snail's endogenous expression in tumor cells 45. Inhibition of the PI3K/Akt signaling pathway could promote E-cadherin expression, reduce Vimentin and Twist expression, restore the polygonal shape of cells, and stimulate mesenchymal-epithelial transformation (MET) 44-47. Furthermore, the PI3K/Akt signaling pathway could also cooperate with other common EMT-related signaling pathways (such as TGF-β 48, NF-κB 49, Ras 50, and Wnt/β-catenin 51, 52) to induce EMT in a direct or indirect mode.

In conclusion, our research demonstrated that NRP1 was upregulated in GC tissues and was associated with a poor prognosis. Moreover, NRP1 induced EMT to enhance the migration and invasion ability of GC cells by activating PI3K/Akt signaling pathway. NRP1 might be a novel prognostic factor and promising therapeutic target for GC patients.

## Figures and Tables

**Table 1:** Correlation between tumor NRP1 expression and clinicopathologic features of the gastric cancer cases

Factors	*n*	NRP1 expression		*P* value
		High (%)	Low (%)	
Age, years				0.2424
<60	137	74	63	
≥60	73	46	27	
Gender				0.4768
Male	126	69	57	
Female	84	51	33	
Tumor size (cm)				< 0.0001
≤5	114	34	80	
>5	96	86	10	
Tumor location				0.8839
Distal	104	58	46	
Body	56	32	24	
Proximal	50	30	20	
Differentiation				0.3174
High	8	3	5	
Moderate	40	19	21	
Poor	116	70	46	
Other	46	28	18	
AJCC TNM stage				< 0.0001
I	44	6	38	
II	43	16	27	
III	96	74	22	
IV	27	24	3	
Depth of invasion				< 0.0001
T1	40	2	38	
T2	18	5	13	
T3	42	23	19	
T4	110	90	20	
Lymph node metastasis				< 0.0001
N0	58	13	45	
N1	43	17	26	
N2	38	25	13	
N3	71	65	6	
Distant metastasis				< 0.0001
Absent (M0)	183	95	88	
Present (M1)	27	25	2	
CEA (preoperative) (ng/ml)				0.2925
≤5	71	37	34	
>5	139	83	56	

**Table 2:** Cox proportional hazard regression analysis for overall survival status in GC cases

Factors	Univariate		Multivariate	
	HR (95% CI)	*P* value	HR (95% CI)	*P* value
Age (years) (<60 vs. ≥60)	0.846 (0.606-1.18)	0.325		
Gender (male vs. female)	0.827 (0.598-1.143)	0.278		
Tumor size (cm) (<5 vs. ≥5)	4.254 (3.013-6.006)	<0.001	0.882(0.605-1.287)	0.516
Differentiation status (High+Moderate vs. Poor+Other)	1.282 (0.884-1.86)	0.19		
Tumor location (Distal vs. Body+Proximal)	1.163 (0.845-1.601)	0.355		
TNM Stage (I+II vs. III+IV)	8.855 (6.402-12.248)	<0.001	4.032(2.416-6.73)	<0.001
T Stage (T1+T2 vs. T3+T4)	14.095 (7.485-26.540)	<0.001	1.945(0.901-4.199)	0.09
N Stage (N0 vs. N1+N2+N3)	6.72 (4.073-11.086)	<0.001	1.595(0.914-2.784)	0.1
Distant metastasis (M0 vs. M1)	29.549 (16.592-52.625)	<0.001	3.569(1.669-7.63)	0.001
NRP1 expression (High vs. Low)	7.056 (4.691-10.614)	<0.001	2.122(1.362-3.305)	0.001
CEA (preoperative) (ng/ml) (≤5 vs. >5)	0.898(0.641-1.26)	0.536		

## Abbreviations

GC: gastric cancer
NRP1: Neuropilin-1
IHC: Immunohistochemistry
OS: overall survival
TCGA: The Cancer Genome Atlas
GTEx: Gene Tissue Expression
EMT: epithelial-mesenchymal transition
SEMA: semaphorin
CCK-8: Cell Counting Kit-8
SDS-PAGE: sodium dodecyl sulfate polyacrylamide gel electrophoresis
PVDF: polyvinylidene difluoride
AJCC: American Joint Committee on Cancer
CEA: carcinoembryonic antigen
IRS: immunoreactive score

## References

[1] Bray F, Ferlay J, Soerjomataram I (2018). Global cancer statistics 2018: GLOBOCAN estimates of incidence and mortality worldwide for 36 cancers in 185 countries. CA Cancer J Clin.

[2] Wadhwa R, Song S, Lee JS (2013). Gastric cancer-molecular and clinical dimensions. Nat Rev Clin Oncol.

[3] Gupta GP, Massague J (2006). Cancer metastasis: building a framework. Cell.

[4] Hanahan D, Weinberg RA (2011). Hallmarks of cancer: the next generation. Cell.

[5] Berx G, van Roy F (2009). Involvement of members of the cadherin superfamily in cancer. Cold Spring Harb Perspect Biol.

[6] He Z, Tessier-Lavigne M (1997). Neuropilin is a receptor for the axonal chemorepellent Semaphorin III. Cell.

[7] Kolodkin AL, Levengood DV, Rowe EG (1997). Neuropilin is a semaphorin III receptor. Cell.

[8] Prud'homme GJ, Glinka Y (2012). Neuropilins are multifunctional coreceptors involved in tumor initiation, growth, metastasis and immunity. Oncotarget.

[9] Soker S, Takashima S, Miao HQ (1998). Neuropilin-1 is expressed by endothelial and tumor cells as an isoform-specific receptor for vascular endothelial growth factor. Cell.

[10] Shi F, Shang L, Yang LY (2018). Neuropilin-1 contributes to esophageal squamous cancer progression via promoting P65-dependent cell proliferation. Oncogene.

[11] Luo M, Hou L, Li J (2016). VEGF/NRP-1axis promotes progression of breast cancer via enhancement of epithelial-mesenchymal transition and activation of NF-kappaB and beta-catenin. Cancer Lett.

[12] Hong TM, Chen YL, Wu YY (2007). Targeting neuropilin 1 as an antitumor strategy in lung cancer. Clin Cancer Res.

[13] Li L, Jiang X, Zhang Q (2016). Neuropilin-1 is associated with clinicopathology of gastric cancer and contributes to cell proliferation and migration as multifunctional co-receptors. J Exp Clin Cancer Res.

[14] Tse BWC, Volpert M, Ratther E (2017). Neuropilin-1 is upregulated in the adaptive response of prostate tumors to androgen-targeted therapies and is prognostic of metastatic progression and patient mortality. Oncogene.

[15] Shi F, Shang L, Pan BQ (2014). Calreticulin promotes migration and invasion of esophageal cancer cells by upregulating neuropilin-1 expression via STAT5A. Clin Cancer Res.

[16] Peng Y, Liu YM, Li LC (2014). MicroRNA-338 inhibits growth, invasion and metastasis of gastric cancer by targeting NRP1 expression. PLoS One.

[17] Tang Z, Li C, Kang B (2017). GEPIA: a web server for cancer and normal gene expression profiling and interactive analyses. Nucleic Acids Res.

[18] He N, Jin Q-n, Huang Y-m (2017). Expression of ADAM8 in gastric carcinoma and its association with clinicopathologic features. Acta Med Univ Sci Technol Huazhong.

[19] Latil A, Bieche I, Pesche S (2000). VEGF overexpression in clinically localized prostate tumors and neuropilin-1 overexpression in metastatic forms. Int J Cancer.

[20] Bachelder RE, Crago A, Chung J (2001). Vascular endothelial growth factor is an autocrine survival factor for neuropilin-expressing breast carcinoma cells. Cancer Res.

[21] Parikh AA, Fan F, Liu WB (2004). Neuropilin-1 in human colon cancer: expression, regulation, and role in induction of angiogenesis. Am J Pathol.

[22] Wang L, Zeng H, Wang P (2003). Neuropilin-1-mediated vascular permeability factor/vascular endothelial growth factor-dependent endothelial cell migration. J Biol Chem.

[23] Soker S, Miao HQ, Nomi M (2002). VEGF165 mediates formation of complexes containing VEGFR-2 and neuropilin-1 that enhance VEGF165-receptor binding. J Cell Biochem.

[24] Hamerlik P, Lathia JD, Rasmussen R (2012). Autocrine VEGF-VEGFR2-Neuropilin-1 signaling promotes glioma stem-like cell viability and tumor growth. J Exp Med.

[25] Bagci T, Wu JK, Pfannl R (2009). Autocrine semaphorin 3A signaling promotes glioblastoma dispersal. Oncogene.

[26] Hu B, Guo P, Bar-Joseph I (2007). Neuropilin-1 promotes human glioma progression through potentiating the activity of the HGF/SF autocrine pathway. Oncogene.

[27] Yaqoob U, Cao S, Shergill U (2012). Neuropilin-1 stimulates tumor growth by increasing fibronectin fibril assembly in the tumor microenvironment. Cancer Res.

[28] Cao Y, Wang L, Nandy D (2008). Neuropilin-1 upholds dedifferentiation and propagation phenotypes of renal cell carcinoma cells by activating Akt and sonic hedgehog axes. Cancer Res.

[29] Liu W, Wu T, Dong X (2017). Neuropilin-1 is upregulated by Wnt/beta-catenin signaling and is important for mammary stem cells. Sci Rep.

[30] Yamamoto M, Suzuki K, Okuno T (2008). Plexin-A4 negatively regulates T lymphocyte responses. Int Immunol.

[31] Chu W, Song X, Yang X (2014). Neuropilin-1 promotes epithelial-to-mesenchymal transition by stimulating nuclear factor-kappa B and is associated with poor prognosis in human oral squamous cell carcinoma. PLoS One.

[32] Ding Z, Du W, Lei Z (2020). Neuropilin 1 modulates TGF-β1-induced epithelial-mesenchymal transition in non-small cell lung cancer. Int J Oncol.

[33] Zeisberg M, Neilson EG (2009). Biomarkers for epithelial-mesenchymal transitions. J Clin Invest.

[34] Scanlon CS, Van Tubergen EA, Inglehart RC (2013). Biomarkers of epithelial-mesenchymal transition in squamous cell carcinoma. J Dent Res.

[35] Smith A, Teknos TN, Pan Q (2013). Epithelial to mesenchymal transition in head and neck squamous cell carcinoma. Oral Oncol.

[36] Mladinich M, Ruan D, Chan CH (2016). Tackling Cancer Stem Cells via Inhibition of EMT Transcription Factors. Stem Cells Int.

[37] Fabregat I, Malfettone A, Soukupova J (2016). New Insights into the Crossroads between EMT and Stemness in the Context of Cancer. J Clin Med.

[38] Lamouille S, Xu J, Derynck R (2014). Molecular mechanisms of epithelial-mesenchymal transition. Nat Rev Mol Cell Biol.

[39] Lee SY, Jeong EK, Ju MK (2017). Induction of metastasis, cancer stem cell phenotype, and oncogenic metabolism in cancer cells by ionizing radiation. Mol Cancer.

[40] Mao Ye, Danning Hu, Lili Tu (2008). Involvement of PI3K/Akt signaling pathway in hepatocyte growth factor-induced migration of uveal melanoma cells. Invest Ophthalmol Vis Sci.

[41] Bakin AV, Tomlinson AK, Bhowmick NA (2000). Phosphatidylinositol 3-kinase function is required for transforming growth factor beta-mediated epithelial to mesenchymal transition and cell migration. J Biol Chem.

[42] Vichalkovski A, Gresko E, Hess D (2010). PKB/AKT phosphorylation of the transcription factor Twist-1 at Ser42 inhibits p53 activity in response to DNA damage. Oncogene.

[43] Silva BS, Yamamoto FP, Pontes FS (2012). TWIST and p-Akt immunoexpression in normal oral epithelium, oral dysplasia and in oral squamous cell carcinoma. Med Oral Patol Oral Cir Bucal.

[44] Lee YJ, Han HJ (2010). Troglitazone ameliorates high glucose-induced EMT and dysfunction of SGLTs through PI3K/Akt, GSK-3beta, Snail1, and beta-catenin in renal proximal tubule cells. Am J Physiol Renal Physiol.

[45] Hong KO, Kim JH, Hong JS (2009). Inhibition of Akt activity induces the mesenchymal-to-epithelial reverting transition with restoring E-cadherin expression in KB and KOSCC-25B oral squamous cell carcinoma cells. J Exp Clin Cancer Res.

[46] Lin YC, Lin JC, Hung CM (2014). Osthole inhibits insulin-like growth factor-1-induced epithelial to mesenchymal transition via the inhibition of PI3K/Akt signaling pathway in human brain cancer cells. J Agric Food Chem.

[47] Kim CJ, Sakamoto K, Tambe Y (2011). Opposite regulation of epithelial-to-mesenchymal transition and cell invasiveness by periostin between prostate and bladder cancer cells. Int J Oncol.

[48] Wenting Xu, Zhen Yang, Nonghua Lu (2015). A new role for the PI3K/Akt signaling pathway in the epithelial-mesenchymal transition. Cell Adh Migr.

[49] Maier HJ, Schmidt-Strassburger U, Huber MA (2010). NF-kappaB promotes epithelial-mesenchymal transition, migration and invasion of pancreatic carcinoma cells. Cancer Lett.

[50] Liao J, Planchon SM, Wolfman JC (2006). Growth factor-dependent AKT activation and cell migration requires the function of c-K(B)-Ras versus other cellular ras isoforms. J Biol Chem.

[51] Sheng S, Qiao M, Pardee AB (2009). Metastasis and AKT activation. J Cell Physiol.

[52] Qiao M, Sheng S, Pardee AB (2008). Metastasis and AKT activation. Cell Cycle.

